# Supplementary material for: Near-field transmission matrix microscopy for mapping high-order eigenmodes of subwavelength nanostructures
Source: Nat Commun. 2020 May 22;11:2575. doi: 10.1038/s41467-020-16263-z (PMC7244505; doi:10.1038/s41467-020-16263-z)
Supplement: Supplementary file 1 — Supplementary Information [file 41467_2020_16263_MOESM1_ESM.pdf]

Supplementary Information

Near-field transmission matrix microscopy for mapping high-order eigenmodes of subwavelength nanostructures

Eunsung Seo, Young-Ho Jin, Wonjun Choi, Yonghyeon Jo, Suyeon Lee, Kyung-Deok Song, Joonmo Ahn, Q-Han Park, Myung-Ki Kim, and Wonshik Choi

Supplementary Note 1: Additional experimental details and results

Detailed experimental setup

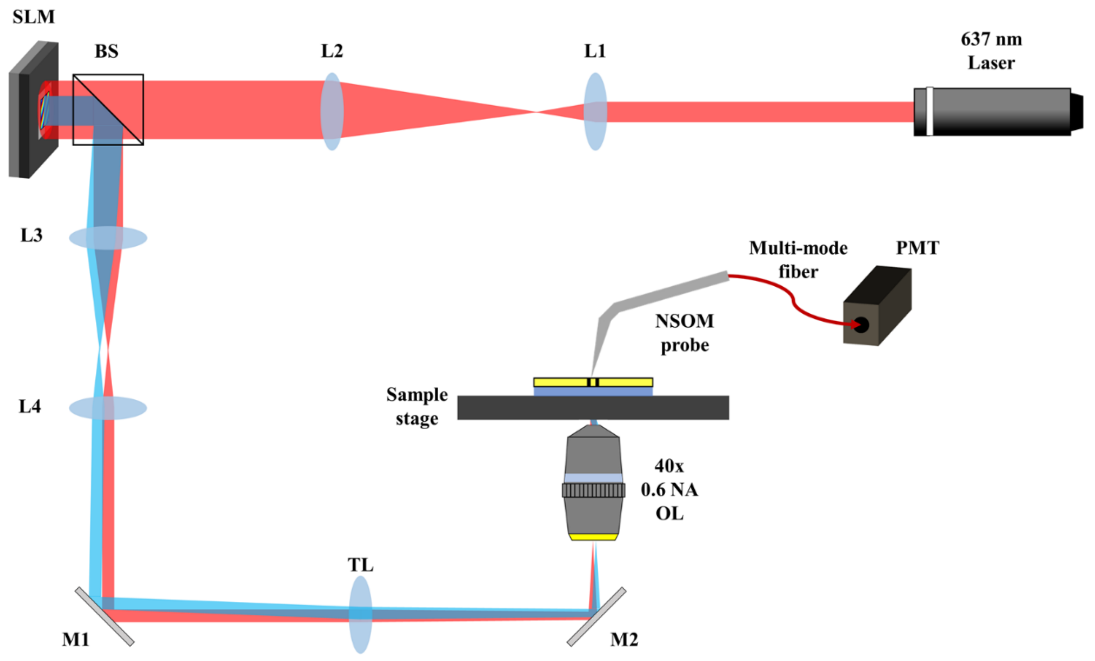

Supplementary Figure 1. Detailed experimental setup. Light source: diode laser with the wavelength of 637 nm and output power of 70 mW. SLM: phase-only spatial light modulator. BS: beam splitter. L1-L4: lenses. M1 and M2: mirrors. OL: 40x objective lens with the numerical aperture of 0.6. PMT: Photomultiplier tube.

To perform the experimental mapping of near-field modes, we combined the far-field phase modulation system with a near-field scanning optical microscope (NSOM, Nanonics MV2000) as shown in Fig. S1. The output beam from a laser diode (Thorlabs inc., LP637-SF70) was enlarged and then collimated to uniformly illuminate a spatial light modulator (SLM, Hamamatsu LCOS-SLM X10468). To measure both the amplitude and phase of the transmitted near-field through the nano-slits, we employed a self-interferometry method as explained in the main text. We wrote a phase pattern on the SLM to generate both the sample and reference waves indicated by respectively the blue and red colored beams in Supplementary Figure 1. These two beams were de-magnified and delivered to the bottom of the NSOM sample stage via an objective lens (OL, Nikon ELWD 40x, 0.6 NA). The overall magnification from the SLM plane to the sample stage was 1/1000x. Near-field waves generated on the upper surface of the nanostructure were measured by scanning the NSOM fiber probe. The diameter of aperture of NSOM probe was 150 nm or 100 nm, and the sample-to-probe distance was maintained within 20 nm. The near-field light captured by the NSOM probe was delivered to the photomultiplier tube (PMT, Hamamatsu, H8259-01).

Coverage of $\mathbf{k}_{\text{in}}$, and the 4-step phase-shifting interferometry

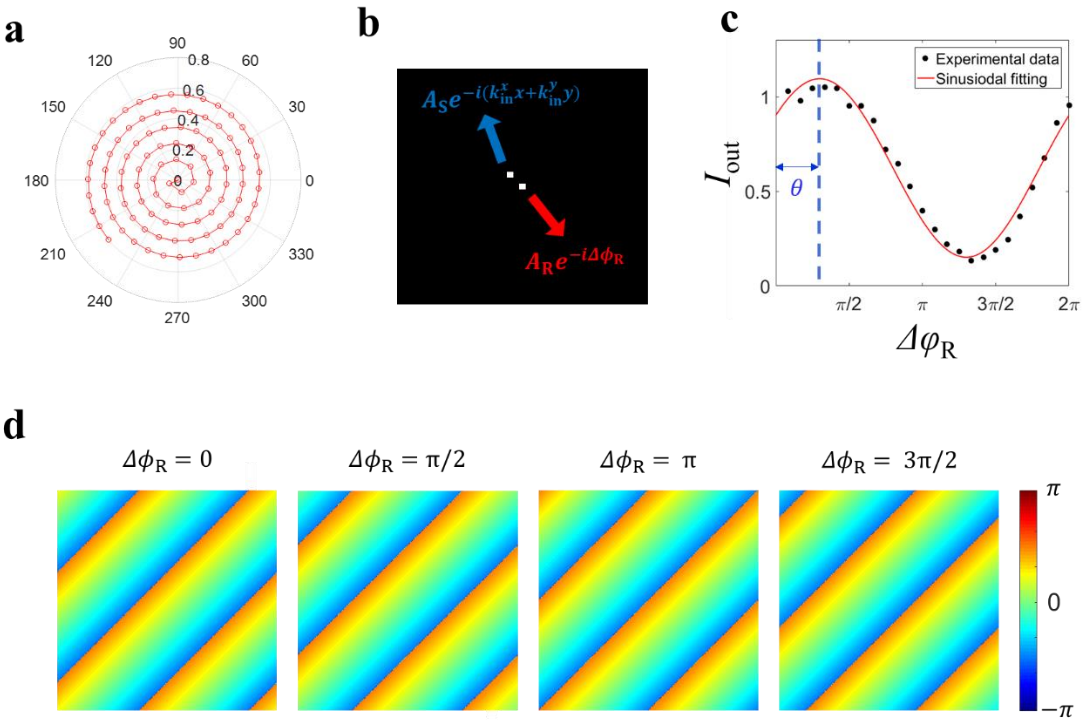

Supplementary Figure 2. Coverage of $\mathbf{k}_{\text{in}}$, and the 4-step phase-shifting interferometry. a, Circular dots indicate those $\mathbf{k}_{\text{in}}$'s covered in the experiment in the coordinate system, $(k_{\text{in}}^x, k_{\text{in}}^y)/k_0$. **b,** 2D map at the Fourier plane of the pattern to be written on SLM. A pixel at the center indicated by a red arrow corresponds to reference wave, and the other indicated by a blue arrow to $\mathbf{k}_{\text{in}}$. **c,** Sinusoidal interference intensity measured by the NSOM probe as the reference phase $\Delta\phi_R$ was scanned from 0 to 2π. Black dots: experimental data, red curve: sinusodial curve fitting. Blue dashed line indicates the relative phase θ between reference and $\mathbf{k}_{\text{in}}$ plane wave at the upper surface of the nanostructure. **d,** Four phase maps written on SLM for the 4-step phase-shitting interferometry at a specific $\mathbf{k}_{\text{in}}$.

As shown in Supplementary Figure 2a, we chose 100 different $\mathbf{k}_{\text{in}}$'s to uniformly cover 0.6 NA objective lens. To identify the phase pattern to be written on SLM, we first mapped each $\mathbf{k}_{\text{in}}$ together with the reference wave of $\mathbf{k}_R = 0$ and $\Delta\phi_R$ at the Fourier plane of the SLM (Supplementary Figure 2b). We then took its inverse Fourier transform and obtained its phase map as shown in Supplementary Figure 2d. This phase map was written on the SLM to simultaneously generate the sample and reference waves. Supplementary Figure 2c shows typical NSOM intensity at a certain probe position as $\Delta\phi_R$ was scanned from 0 to 2π, from which we could determine the amplitude and phase of the near-field wave with respect to reference wave. To minimize the number of measurements in the matrix measurements, we reduced the number of phase steps to four, i.e. $\Delta\phi_R$=0,$\frac{\pi}{2}$,π and $\frac{3\pi}{2}$. By using the four measured values of near-field intensity, we could obtain the near-field complex-field amplitude for the corresponding $\mathbf{k}_{\text{in}}$ and the probe position by using a phase-shifting interferometry algorithm. Supplementary Figure 2d shows the example of the four phase patterns written on SLM for $\Delta\phi_R$'s in multiples of $\frac{\pi}{2}$.

Double nano-slits rotated clockwise by $\theta = 14^o$

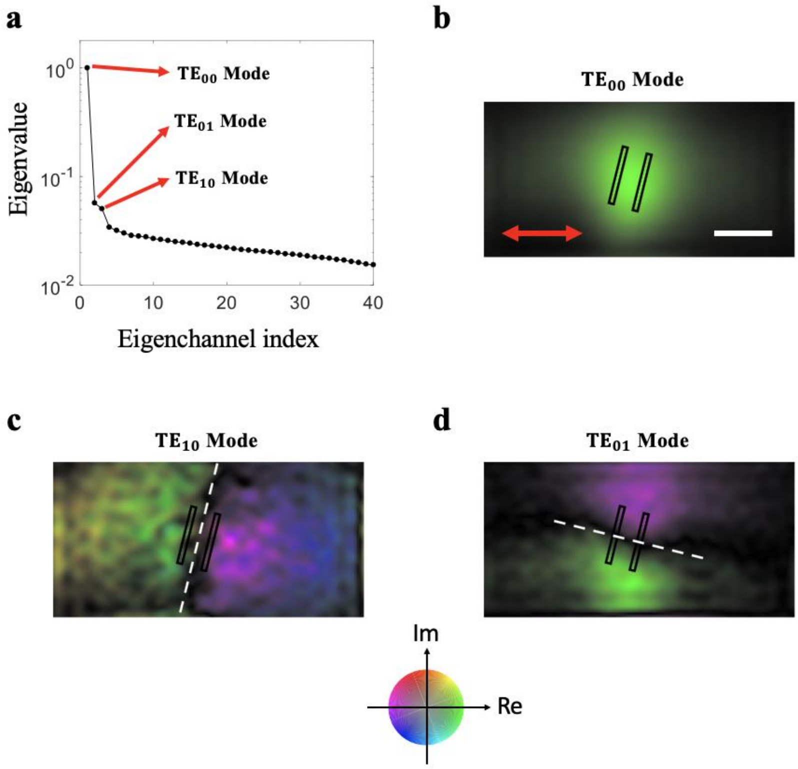

Supplementary Figure 3. Near-field eigenmodes of the double nano-slits rotated by 14° clockwise. a, Eigenvalue distribution by performing the singular value decomposition of the experimentally measured FNTM. **b,** Complex field map of TE_{00} mode. Red arrow indicates the direction of linear polarization of the far-field illumination. Scale bar, 150 nm. **c** and **d,** Complex field maps of TE_{10} mode and TE_{01} mode, respectively. White dashed lines indicate the phase jumps of each mode. Estimated amount of rotation angle was 14 degrees in the clockwise direction.

The higher-order modes shown in Fig. 2 of the main text depends on the rotation angle of the double nano-slits. To verify this, we performed additional experiments for another arbitrary rotation angle of the sample. Supplementary Figure 3a shows the eigenvalue distribution of the FNTM. First eigenmode was TE_{00} mode as shown in Supplementary Figure 3b. Supplementary Figures 3c and 3d show the near-field maps of the two other eigenmodes, i.e. TE_{10} and TE_{01} modes. TE_{10} mode was transverse antisymmetric mode and TE_{01} mode is the first-order longitudinal mode along the long axis of the nano-slits. From the angles of the dark lines due to the phase jumps and intensity nodes of TE_{10} and TE_{01} modes indicated by the white dashed lines, the rotation angle of the sample was measured to be 14 degrees, which agrees well with the sample preparation. Because of the orthogonality between TE_{10} and TE_{01}, two dashed lines were perpendicular with respect to each other. In comparison with the data shown in Fig. 2 of the main text, higher-order longitudinal modes were below the noise level. This is because the excitation of longitudinal modes was less efficient at the smaller rotation angle.

Near-field eigenmodes of double nano-slits depending on the scanning step of the NSOM probe

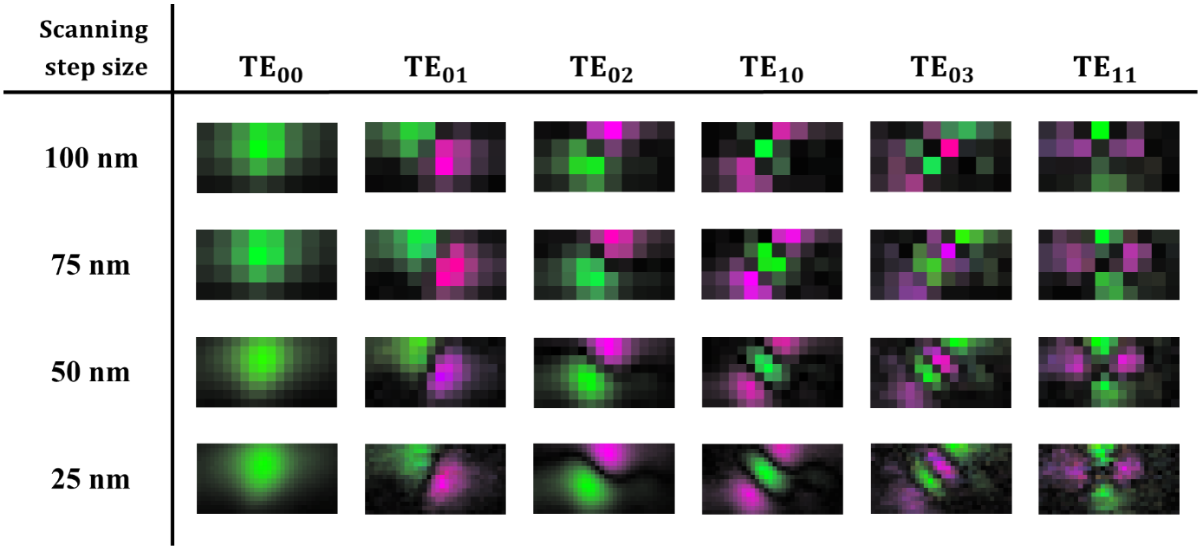

Supplementary Figure 4. Near-field eigenmodes of double nano-slits depending on the scanning step of the NSOM probe. Eigenmodes for the scanning steps of 100 nm, 75 nm, 50 nm, and 25 nm are shown. The white rectangles outline the boundaries of the nano-slits. Scale bar, 150 nm.

Similar to the analysis for the number of illumination angles shown in Fig. 3, we conducted mode mapping analysis depending on the scanning step size (Supplementary Figure 4). We constructed FNTMs with the scanning step of 50 nm, 75 nm and 100 nm from the original FNTM taken at 25 nm scanning step and obtained their respective eigenmodes. Up to the 50 nm scanning step, the nodes of the higher order modes were well visible. However, the image pixelation became so pronounced with the further increase in the scanning step that the fine mode structures were lost, especially at higher order modes. Considering the trade-off relation between the acquisition time and scanning step size, the scanning step of 50 nm can also be a good choice because the matrix acquisition time can be reduced by a factor of four.

One-dimensional near-field mode mapping depending on the slit gap

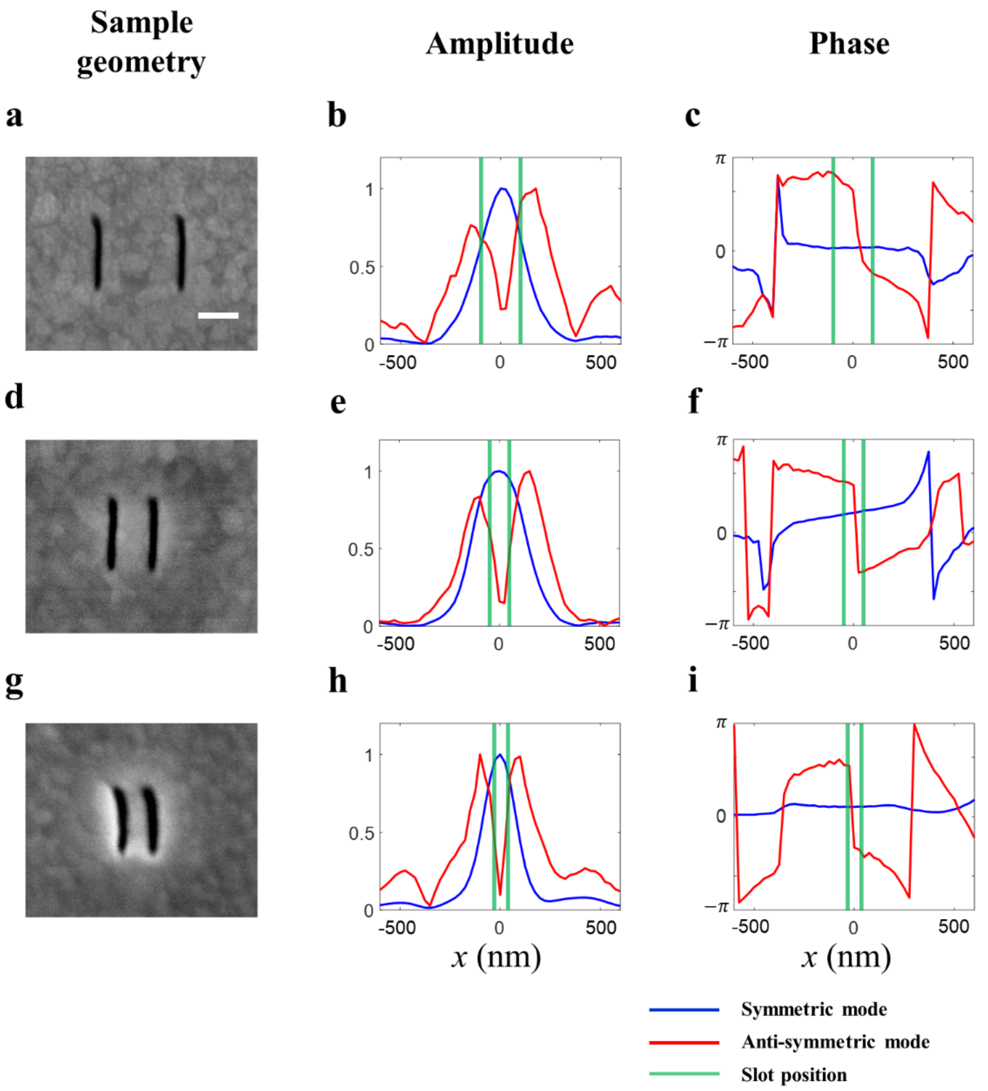

Supplementary Figure 5. One-dimensional near-field eigenmodes mapping for various gap sizes between the nano-slits. a, d and **g,** Scanning electron microscope images of double nano-slits with gap sizes D = 180 nm, 80 nm and 50 nm, respectively. Scale bar, 100 nm. **b, e,** and **h** show the amplitude profiles of the near-field eigenmodes, and **c, f,** and **i** the phase profiles. Blue and red curves indicate symmetric and antisymmetric modes, respectively, and the green vertical bars indicate the positions of nano-slits.

We investigated the resolving power of antisymmetric modes depending on the slit gap size. We prepared three pairs of nano-antennas with various gaps (D = 180 nm, 80 nm, and 50 nm) and measured the FNTM for each case. By using singular value decomposition of transmission matrix, we obtained near-field eigenmodes. As shown in Supplementary Figure 5, near-field mode mapping method was working for nano-slits with various gap sizes. The smallest gap size at which antisymmetric mode could be identified experimentally was 50 nm in our system when the 150-nm-diameter probe aperture was used. The theoretically predicted minimum gap size at which the same probe can reveal the antisymmetric mode is 20 nm, but this was out of reach in the present experiment due to the fabrication limit.

Resolving multiple nano-slits neighboring closer than the probe aperture diameter

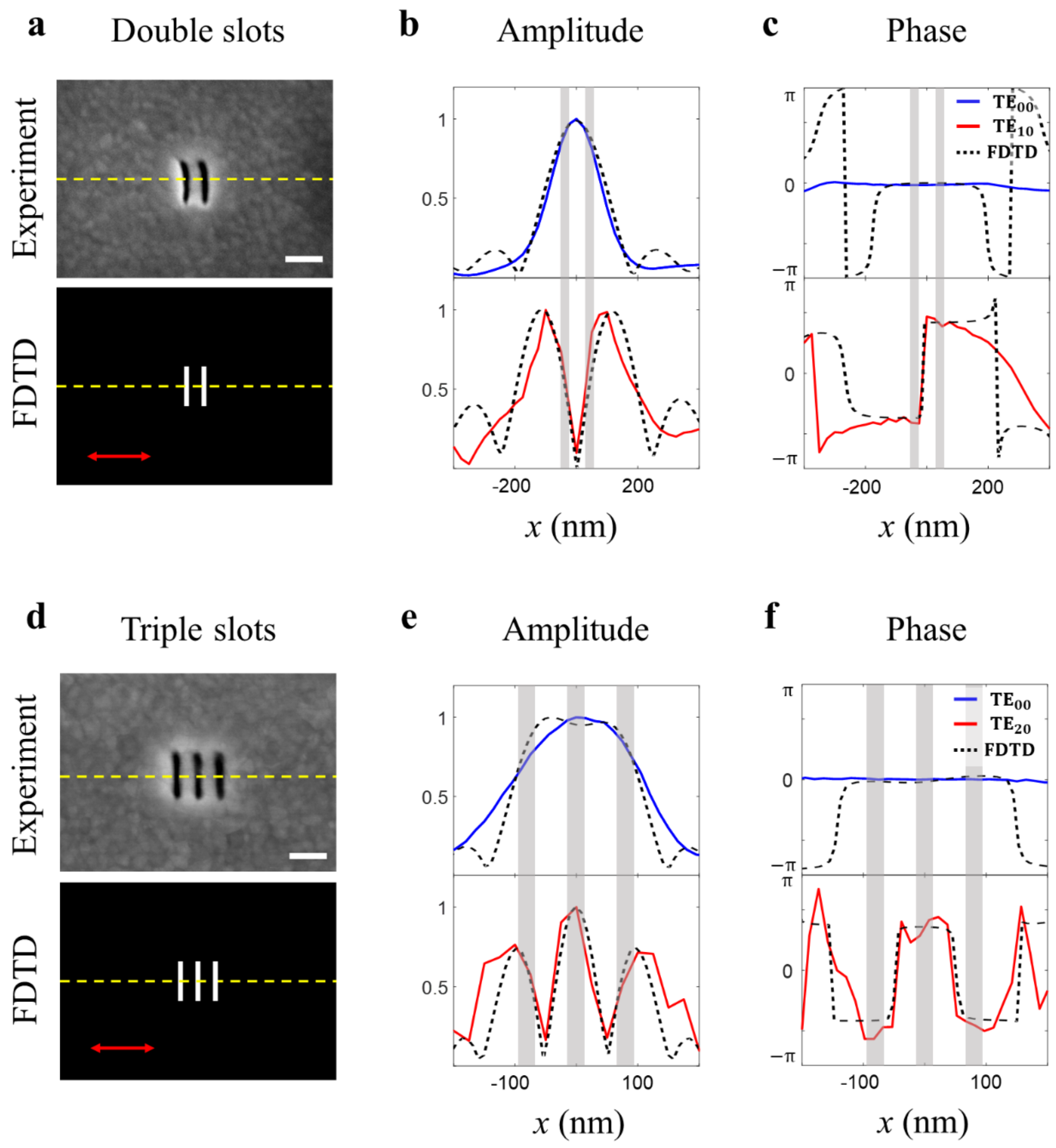

Supplementary Figure 6. Resolving multiple slits whose gap is smaller than the aperture diameter. **a-c,** Near-field mode mapping of the double nano-slits with a gap of 50 nm and width of 20 nm. **a.** Scanning electron micrograph of the sample, **b,** amplitude profiles and **c,** phase profiles of the eigenmodes along the yellow dashed line in **a.** Blue and red curves are the profiles of TE_{00} and TE_{10} modes, respectively, from experiment. The FDTD simulation results are shown as black dashed curves. The vertical gray bars indicate the positions and widths of the nano-slits. Bottom image in **a** shows the numerically prepared sample. The red arrow indicates the polarization of the far-field illumination. Scale bar, 150 nm. **d-f** Same as **a-c**, but for the triple nano-slits with a gap of 50 nm gap and width of 30 nm. To resolve the triple nano-slits, we obtained the TE_{20} mode represented by the red curves instead of the TE_{10} mode.

The identification of the antisymmetric mode provides an unusual opportunity to resolve the nano-slits beyond the limit set by the physical size of the NSOM probe aperture. To investigate the resolving power of the near-field eigenmode mapping method, we fabricated double and triple nano-slits whose gap was 50 nm, much smaller than the probe aperture. The direction of polarization of the incident wave was set orthogonal to the long axes of the slits. The FNTM was measured by scanning the NSOM probe

through the centers of the slits, indicated by the yellow dashed line in Supplementary Figure 6a. In the experiments with the double nano-slits, the diameter of the probe aperture was 150 nm, and the probe scanning step was 25 nm. Supplementary Figures 6b and 6c show respectively the amplitude and phase profiles of the transverse near-field modes obtained by the experimentally measured FNTM for the double nano-slits shown in the upper image of Supplementary Figure 6a. The gray vertical bars indicate the positions of the nano-slits. The symmetric mode (TE_{00} mode, blue curves) did not reveal the existence of the two slits as they were driven in phase. On the contrary, the antisymmetric mode (TE_{10} mode, red curves) clearly resolved the two slits. The position where the destructive interference of the near-field modes between the two nano-slits occurred was distinct from the sharp amplitude dip in Supplementary Figure 6b and steep phase jump in Supplementary Figure 6c. This position exactly matches with the center of the two slits, which is a clear evidence that the identification of the antisymmetric mode greatly enhanced the resolving power. To support the experimental data, we performed numerical simulations using the FDTD method for the numerically prepared sample (bottom image of Supplementary Figure 6a) having the same configuration as the experiment (see Supplementary Note 3 for details). The black dashed lines in Supplementary Figures 6b and 6c show the amplitude and phase profiles of the near-field eigenmodes obtained by the FNTM calculated by the FDTD simulations, respectively. The excellent agreement between the experiments and simulation results supports the validity of our experiments. The discrepancies in the phase profile at a distance from the nano-slits are not critical as the amplitude there is small.

The identification of the antisymmetric mode enabled us to locate the center position between the two nano-slits, which is similar to finding the center of the point-spread-function in far-field imaging. In this respect, this may not be sufficiently general to claim the resolving power. Therefore, we considered imaging of triple nano-slits to evaluate whether the proposed method can resolve the two neighboring center positions of the three nano-slits. In the experiments with the triple nano-slits, the diameter of the probe aperture was 100 nm, and the probe scanning step was 15 nm. Supplementary Figures 6e and 6f show the transverse near-field modes of the triple nano-slits for the sample shown in the upper image of Supplementary Figure 6d. The blue and red curves represent the profiles of the symmetric and TE_{20} modes, respectively. Similar to the double nano-slits, the amplitude dips and steep phase jumps of the TE_{20} mode were located at the centers of the two neighboring slits because the phase difference between the adjacent slits was close to π. This led to resolving triple nano-slits whose gap size was two times smaller than the aperture diameter. These results further show the excellent agreement with the near-field modes obtained by the FDTD simulation, as indicated by the black dashed lines in Supplementary Figures 6e and 6f. The theoretical limit of the resolving power is determined by the diameter of probe aperture and sensitivity of the NSOM system. The finite scanning step of NSOM probe and the phase difference between the neighboring nano-slits are additional factors affecting to the resolving power. According to our analysis, the smallest gap that our system with 150 nm probe aperture can resolve in theory is 20 nm, but this was out of reach due to the fabrication limit of sample preparation. Further improvement of the sensitivity of the system and the adoption of advanced fabrication technology are expected to enhance the resolving power.

Supplementary Note 2: Theoretical model for extracting high-order eigenmodes

Interpretation of the singular value decomposition

As the interaction of incident light with the multiple nano-slits is a linear process, we can consider the near-field complex-field maps $E_S(x, y; \mathbf{k}_{in})$ as the superposition of the orthogonal near-field eigenmodes TE_{nm}. Let us represent the near-field eigenmodes as $f_p(x, y)$ at the upper surface of the sample, where p indicates (n, m). Then the column of the FNTM obtained by the specific incident wavevector $\mathbf{k}_{in}^l$ can be written in terms of the orthogonal near-field eigenmodes:

$$t(x, y; \mathbf{k}_{in}^l) = \sum_p a_{lp} f_p(x, y). \quad \text{(S1)}$$

After the SVD, each column vector of V contains the coefficients to superpose $t(x, y; \mathbf{k}_{in}^l)$ in such a way to obtain each $f_p(x, y)$. For instance, the multiplication of the qth column of V to the FNTM results in the qth eigenmode $f_q(x, y)$ weighted by the singular value τ_q, i.e.

$$\sum_l t(x, y; \mathbf{k}_{in}^l) V_{lq} = \sum_l \sum_p a_{lp} V_{lq} f_p(x, y) = \tau_q f_q(x, y). \quad \text{(S2)}$$

In fact, in the transmission matrix decomposed by SVD, $t(x, y; \mathbf{k}_{in}^l) = U\Sigma V^\dagger$, the multiplication of qth column vector of V to t results in the qth column vector of U multiplied by τ_q. Therefore, the qth column of U corresponds to $f_q(x, y)$.

A simple double-slit model

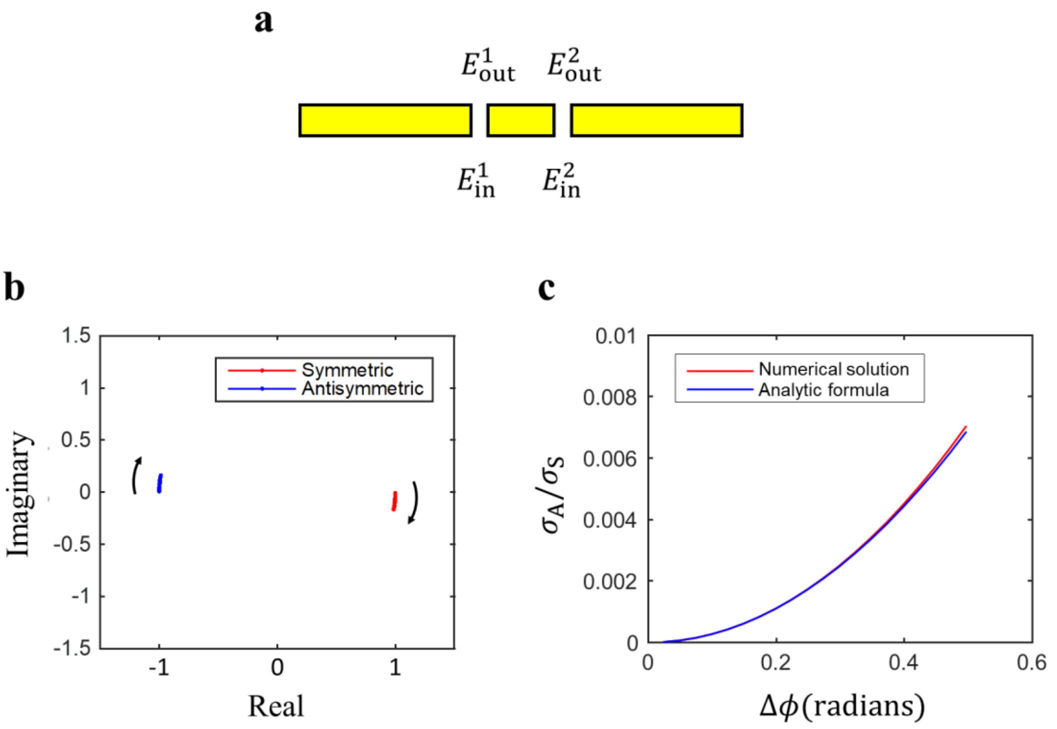

Supplementary Figure 7. A simple double-slit model. a, Geometrical description of coupling incident electric field (E_{in}^1, E_{in}^2) to the output electric field (E_{out}^1, E_{out}^2). Slit on the left is labeled as 1, and the other slit as 2. **b,** Eigenvectors, $\mathbf{v}_1 = (a_1, b_1)$ and $\mathbf{v}_2 = (a_2, b_2)$, that correspond to symmetric and antisymmetric modes, respectively. a_1/b_1(red) and a_2/b_2(blue) are plotted as $\Delta\phi$ is increased from 0 to 0.5 for $\frac{K}{J} = 0.2$ The black arrows indicate the increasing directions of $\Delta\phi$. **c,** Eigenvalue ratio σ_A/σ_S as a function of $\Delta\phi$. Red and blue curves are obtained from numerical calculations and analytic calculation, respectively.

The identification of an antisymmetric mode by the singular value decomposition of the FNTM can be understood by a simple double-slit model. The transmission of incident field through the double-slit system can be described by the following equation:

$$\begin{pmatrix} E_{\text{out}}^1 \\ E_{\text{out}}^2 \end{pmatrix} = \begin{pmatrix} J & K \\ K & J \end{pmatrix} \begin{pmatrix} E_{\text{in}}^1 \\ E_{\text{in}}^2 \end{pmatrix}. \tag{S3}$$

Here, the vectors $(E_{\text{in}}^1, E_{\text{in}}^2)$ and $(E_{\text{out}}^1, E_{\text{out}}^2)$ indicate the electric fields at the input and output planes, respectively, and subscripts 1 and 2 stand for the left- and right-hand slits, respectively. J describes the coupling constant to the output of the same slit as the input, and K the coupling to the other slit's output. In the experiment, we cannot directly measure J and K by the far-field excitation because the slit separation is too small for the far-field illumination to excite individual slits. Likewise, the symmetric and antisymmetric modes cannot be individually addressed by the far-field excitation. In other words, far-field excitation cannot separately excite two orthogonal modes of the double-slit system. Instead, the combination of orthogonal modes is measured in the experiment, and SVD serves as means to identify the orthogonal modes from the superposed measurements.

For a given far-field incident wavevector $\mathbf{k}_{\text{in}}$, the phase difference of incident wave between the two slits is given by $\Delta\phi(\mathbf{k}_{\text{in}}) = |\mathbf{k}_{\text{in}} \cdot \mathbf{D}|$ as explained in the main text with $\Delta\phi \leq \frac{2\pi D}{\lambda}$ ~0.5, much smaller than π. Therefore, the incident wave is mostly coupled to the symmetric mode even at the maximum incidence angle. The incident electric field at the two slits can be written as $(E_{\text{in}}^1, E_{\text{in}}^2) = (E_0, E_0 e^{-i\Delta\phi(\mathbf{k}_{\text{in}})})$ for any given $\mathbf{k}_{\text{in}}$. We sent 100 different incident wavevectors in the experiment, but for simplicity let's consider two representative incident wavevectors, one with normal illumination and the other with the phase differences of $\Delta\phi$. Then, the recorded output fields are respectively written as

$$\begin{pmatrix} E_{\text{out}}^1 \\ E_{\text{out}}^2 \end{pmatrix} = \begin{pmatrix} J & K \\ K & J \end{pmatrix} \begin{pmatrix} E_0 \\ E_0 \end{pmatrix} = \begin{pmatrix} JE_0 + KE_0 \\ KE_0 + JE_0 \end{pmatrix} \tag{S4}$$

And

$$\begin{pmatrix} E_{\text{out}}^1 \\ E_{\text{out}}^2 \end{pmatrix} = \begin{pmatrix} J & K \\ K & J \end{pmatrix} \begin{pmatrix} E_0 \\ E_0 e^{-i\Delta\phi} \end{pmatrix} = \begin{pmatrix} JE_0 + KE_0 e^{-i\Delta\phi} \\ KE_0 + JE_0 e^{-i\Delta\phi} \end{pmatrix}. \tag{S5}$$

Using these two output vectors, we construct the following far- to near-field transmission matrix, where SVD is applied, i.e.

$$T = E_0 \begin{pmatrix} J + K & J + Ke^{-i\Delta\phi} \\ K + J & K + Je^{-i\Delta\phi} \end{pmatrix} = U\Sigma V^+. \tag{S6}$$

We obtain two eigenvectors $\mathbf{v}_\text{S} = (a_1, b_1)$ and $\mathbf{v}_\text{A} = (a_2, b_2)$ from the columns of U, and their associated eigenvalues, σ_S and σ_A, from the diagonal elements of the matrix Σ^2. Figure S7b shows the plot of a_1/b_1 and a_2/b_2, from which we could see that $\mathbf{v}_\text{S} \approx \frac{1}{\sqrt{2}}(1,1)$ and $\mathbf{v}_\text{A} \approx \frac{1}{\sqrt{2}}(1,-1)$, i.e. they correspond to symmetric and antisymmetric modes, respectively. This confirms that SVD of the measured FNTM leads to the mapping of antisymmetric mode as well as symmetric mode. In Supplementary Figure 7c, the ratio between the eigenvalues, $\sigma_\text{A}/\sigma_\text{S}$ is plotted. The eigenvalue of the antisymmetric mode is two or three orders of magnitude smaller than that of the symmetric mode. This is mainly because the far-field excitations mostly drive the symmetric mode due to the small input phase difference $\Delta\phi$. This is confirmed by the increase of $\sigma_\text{A}/\sigma_\text{S}$ as we increase $\Delta\phi$. However, even with the use of largest illumination angle, i.e. $|\mathbf{k}_{\text{in}}| = k_0$, $\sigma_\text{A}/\sigma_\text{S}$ is only 0.007. This explains why it is difficult to observe antisymmetric mode in the conventional NSOM imaging. In fact, we can analytically derive the following eigenvalue ratio in the case when $\Delta\phi \ll 1$, which is plotted as a blue curve in Supplementary Figure 7c.

$$\frac{\sigma_\text{A}}{\sigma_\text{S}} = \frac{(1-K/J)^2}{16(1+K/J)^2} \Delta\phi^2 \tag{S7}$$

We could observe good agreements with the exact solution by the numerical simulation (red curve in Supplementary Figure 7c). In the weak-coupling regime ($K \ll J$), the eigenvalue ratio is approximately given as $\sigma_\text{A}/\sigma_\text{S} \approx \Delta\phi^2/16$.

Simulated transmission matrix analysis

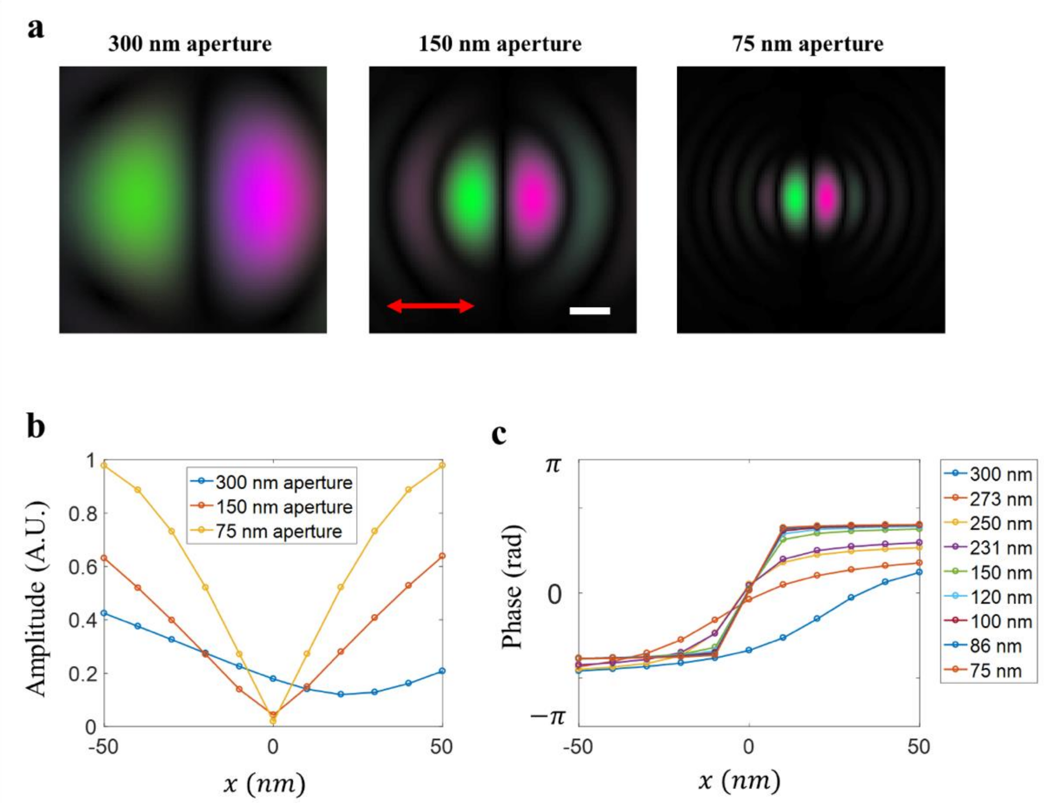

Supplementary Figure 8. Antisymmetric modes depending on the probe aperture size. a, Complex-field maps of the antisymmetric modes for the detection NA of k_0, $2k_0$ and $4k_0$. Scale bar, 150 nm. **b** and **c,** Amplitude and phase profiles of the antisymmetric modes for various diameters of probe apertures.

While the analytic double-slit model in Supplementary Note 2 predicts the eigenvalue ratio between symmetric and antisymmetric modes, it cannot describe the spatial mode profiles of the eigenmodes. Here, we developed an analysis method that can accommodate the effect of the probe aperture size. Using FDTD method, we computed the near-field complex-field map $E^{\mathrm{S}}(x,y)$ for a single nano-slit at the output plane in the case of normal far-field illumination from the bottom of the sample. The pixel resolution of the near-field map was set 10 nm. For an arbitrary incident wave $\left(E_{\mathrm{in}}^{1}, E_{\mathrm{in}}^{2}\right)$, the output field for the double nano-slits can then be written as

$$E_{\mathrm{out}}(x,y;\Delta\phi) = E_{\mathrm{in}}^{1}E^{\mathrm{S}}(x-D/2,y) + E_{\mathrm{in}}^{2}E^{\mathrm{S}}(x+D/2,y) \qquad \text{(S8)}$$

in the weak coupling regime, where D is the separation between the two slits. Similar to the simple double-slit model, we control incident wave $\left(E_{\mathrm{in}}^{1}, E_{\mathrm{in}}^{2}\right) = \left(E_0, E_0 e^{-i\Delta\phi}\right)$ with $\Delta\phi$ determined by $\mathbf{k}_{\mathrm{in}}$ and compute the output field for the same set of $\mathbf{k}_{\mathrm{in}}$ used in the experiment. We then construct a FNTM and obtain its near-field eigenmodes by applying SVD. Since we now have the spatial dependence of the output field, we can incorporate the effect of the finite aperture size.

NSOM probe aperture serves as a low-pass filter. For a given aperture size α, the maximum spatial frequency that the probe can capture is given by

$$k_{\mathrm{max}} = \frac{\lambda}{2\alpha}k_0. \qquad \text{(S9)}$$

For instance, when α is equal to the far-field diffraction limit, i.e. $\lambda/2$, then the maximum spatial frequency is given by k_0. Since the wavelength of light source used for FDTD simulation is 637 nm, the aperture size of 300 nm corresponds almost to $k_{\mathrm{max}} = k_0$. Likewise, aperture diameters of 150 nm and 75 nm correspond to k_{max} of $2k_0$ and $4k_0$, respectively. In constructing a FNTM, we apply low-

pass filtering to the output field calculated by Eq. (S8) with the maximum spatial frequency set by $k_{\max}$, which is given by the aperture probe size α. Supplementary Figure 8a shows the antisymmetric modes obtained from the low-pass filtered FNTMs for the aperture diameters of 300 nm, 150 nm and 75 nm for the case of D = 50 nm. We observed that the size of overall mode shape is smaller and the phase jump becomes sharper as the size of aperture probe is made smaller.

Supplementary Figures 8b and 8c show respectively the amplitude and phase profiles of the antisymmetric modes depending on the probe aperture size. The precision to resolve the center of the two slits was good enough when the aperture size of NSOM probe was 150 and 75 nm. In fact, the phase variation around the center amounts to almost π when the aperture size was equal or smaller than 150 nm. In the case of 300 nm aperture, however, the amplitude node and phase jump were no longer at the center of the double slits. Additionally, the phase profile was too smoothened to identify the center of the double nano-slits. These results imply that probe aperture as small as 150 nm is necessary to properly resolve antisymmetric mode of double nano-slits whose gap size is 50 nm.

Supplementary Note 3: Numerical Analysis using FDTD simulation

Numerical mode analysis for the double nano-slits

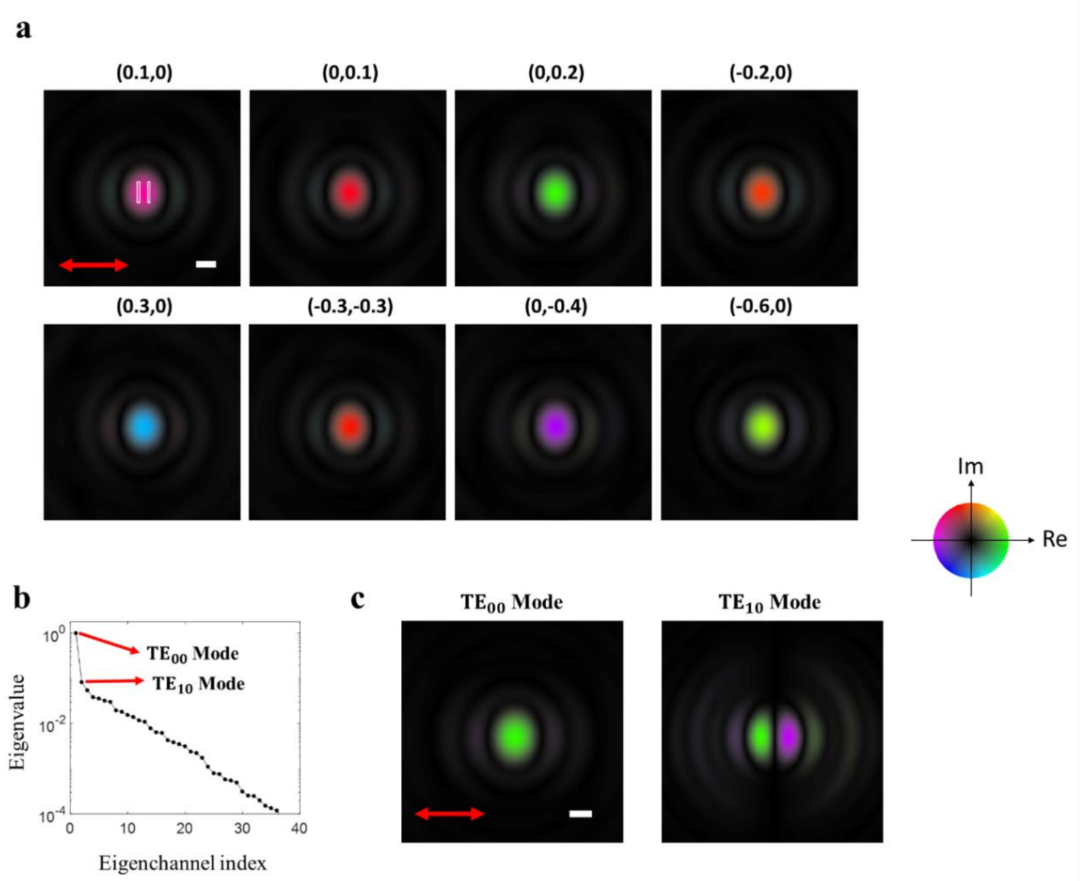

Supplementary Figure 9. FDTD simulation for a FNTM of double nano-slits. a, Complex-field maps of near-field waves obtained at 10 nm above the surface of the nano-slits for various $\mathbf{k}_{\text{in}}$'s. The coordinate above each sub-figure indicates $\mathbf{k}_{\text{in}}$ in unit of k_0. **b,** Eigenvalues of the transmission matrix sorted in the descending order after normalizing them by the largest eigenvalue. **c,** Complex-field maps of TE_{00} and TE_{10} transverse modes. Scale bar, 150 nm. Color map, real and imaginary values of the near-field wave.

We conducted a numerical simulation using FDTD method to verify the experimental observation of near-field eigenmode mapping. Simulation condition was set almost equal to that of the experiments. The same set of $\mathbf{k}_{\text{in}}$ used in the experiments were numerically generated and sent from the bottom of the double nano-slits engraved on a 100 nm-thick gold film. The slit gap was set 50 nm, and the real and imaginary dielectric constants of gold were set $\varepsilon_{\text{real}}$= -12.33, $\varepsilon_{\text{imag}}$= 2.09, respectively. Numerical mesh grid size was $\Delta x = \Delta y = \Delta z$ =10 nm. For each $\mathbf{k}_{\text{in}}$, we computed the transmission of far-field illumination through the nano-slits in the steady state and recorded the transmitted near-field wave at 10 nm above the gold surface. The acquired field was low-pass filtered by the maximum spatial frequency of $2k_0$, which corresponds to the sampling by 150 nm-diameter probe aperture. Supplementary Figure 9a shows the complex-field maps of the computed near-field waves for various $\mathbf{k}_{\text{in}}$, which were similar to those measured in the experiment (Fig. 1c of the main text). Using this set of measurements, we constructed a FNTM in the same way as the experiment and obtained its eigenvalue distribution as shown in Supplementary Figure 9b. The first two eigenvalues correspond to TE_{00} and TE_{10} modes, and their respective eigenmode maps are shown in Supplementary Figure 9c. As we discussed in Fig. 3a of the main text, FDTD simulation results were in excellent agreement with the experimental data, thereby supporting the validity of our experiment.

The near-field phase map for the normal illumination of reference beam

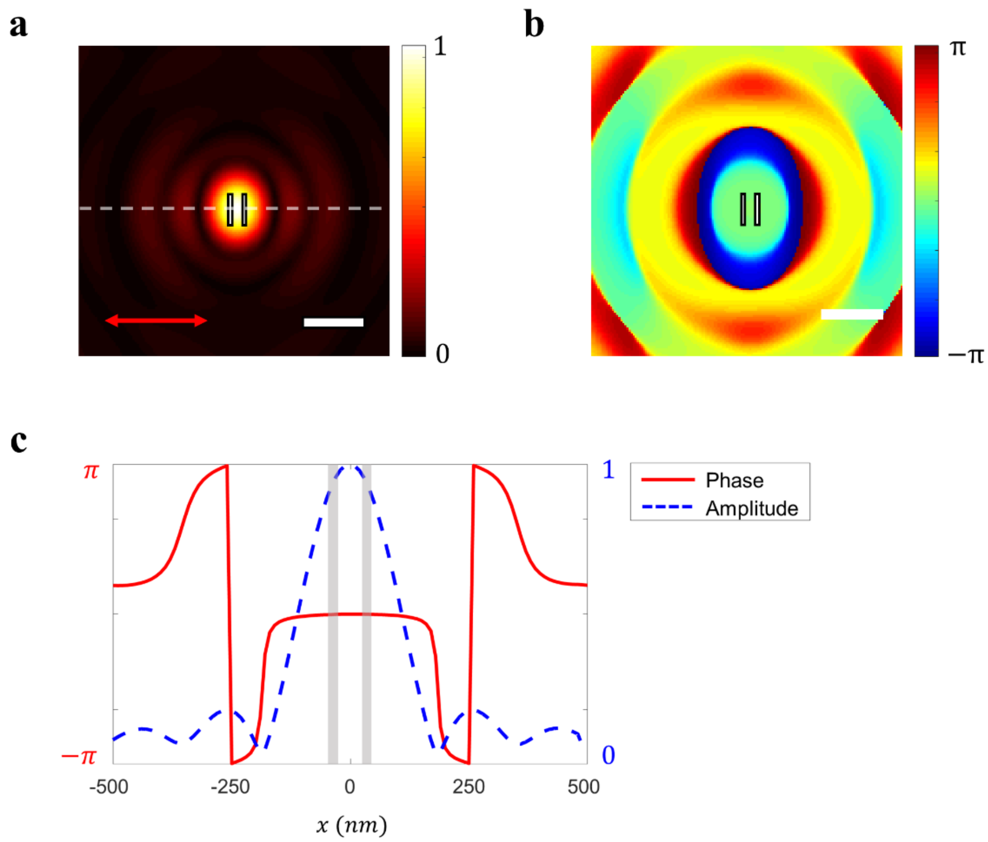

Supplementary Figure 10. Electric field distribution for normal illumination of the reference wave. a and **b,** Normalized amplitude map and the phase map of the near-field wave at the surface of the sample, respectively. **c,** Line profiles of amplitude and phase along the transverse direction of double nano-slits indicated as white dashed line in **a**. Scale bar, 200 nm.

We conducted a numerical simulation to show that the phase of reference wave is spatially flat across the double nano-slits. Supplementary Figures 10a and 10b show the normalized amplitude map and phase map of the near-field wave at the sample surface, respectively. And we obtained line profiles of amplitude and phase along the white dashed line indicated in Supplementary Figure 10a. As shown Supplementary Figure 10c, the phase of near-field for the normally incident reference wave is flat in the range of gaussian shape of the amplitude.

The effect of tip-sample interaction depending on the tip-sample distance

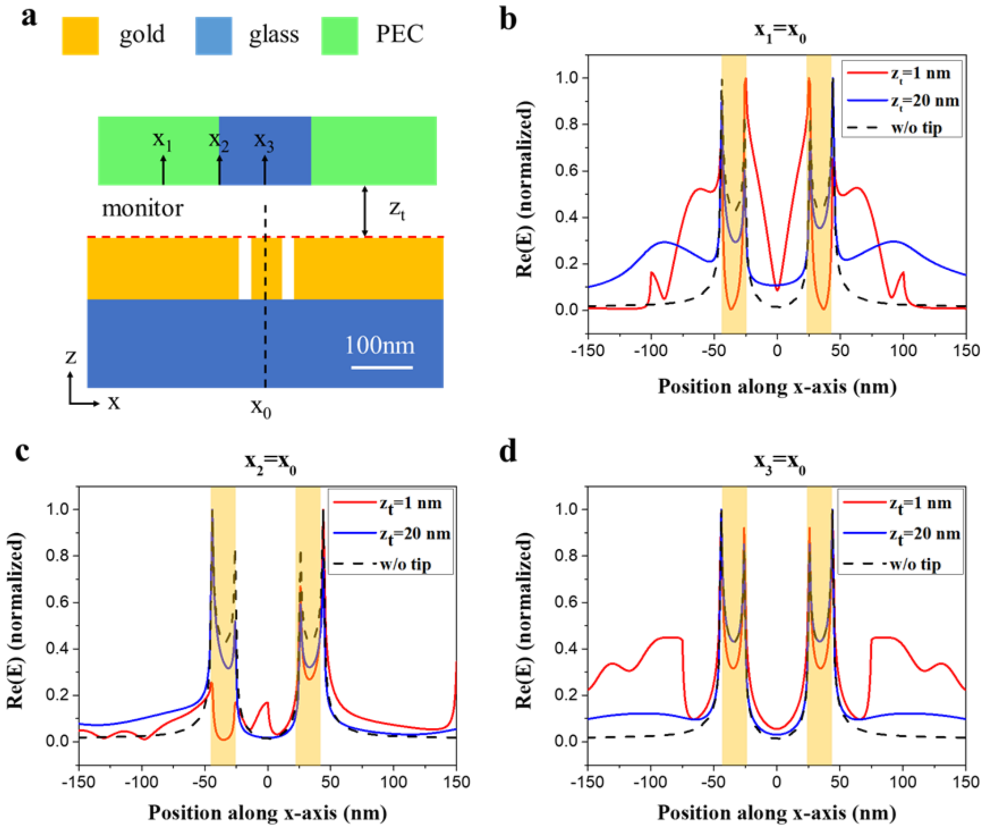

Supplementary Figure 11. The effect of tip-sample interaction. a, Simulation layout for various tip-sample geometries. **b-d,** The E-field profile on gold surface when tip is either 1 nm (red curve) or 20 nm (blue curve) above the sample for the cases when the center of the metal coating (x_1), the fiber-metal boundary of aperture (x_2), and the center of the aperture (x_3), are matched to the center of the double nano-slits (x_0), respectively. Black dashed curves are the near-field profiles in the absence of the tip.

We verified that the tip-sample interaction in our experiment was negligible from the good agreements between the experimentally identified eigenmodes with those acquired by the FDTD simulations (Supplementary Figure 5). Here, we conducted additional FDTD simulations and confirmed that the effect of tip-sample interaction is negligible for the tip-sample distance of 20 nm, while the modification of the near-field is significant for the tip-sample distance of 1 nm (Supplementary Figure 11).

The layout of the sample geometry for the additional FDTD simulation is shown in Supplementary Figure 11a. The NSOM aperture tip was modeled as a PEC-glass-PEC slab. We considered two cases of the tip-sample distance z_t, i.e. z_t =1 nm and 20 nm. And the relative lateral position of the tip to the double nano-slits was varied. Figures S11b-d show the electric field at the sample surface (red dashed line in Supplementary Figure 11a) when the center of the metal coating (x_1), the fiber-metal boundary of aperture (x_2), and the center of the aperture (x_3) are matched to the center of the sample (x_0), respectively. For comparison, the near-field map at the sample surface is shown in the absence of the tip (black dashed curves).

The near-field was disturbed by the tip when the tip-sample distance is 1 nm for all three cases. In particular, the disturbance was pronounced when the metal side of the tip was positioned above nano-slits (Supplementary Figures 11b and 11c). On the contrary, when the tip-sample distance is 20 nm, the near-field maintains a similar shape to that without the tip for all the cases. These simulation results support that the tip-sample interaction was not significant in our experiments.

Supplementary Note 4: Sample fabrication

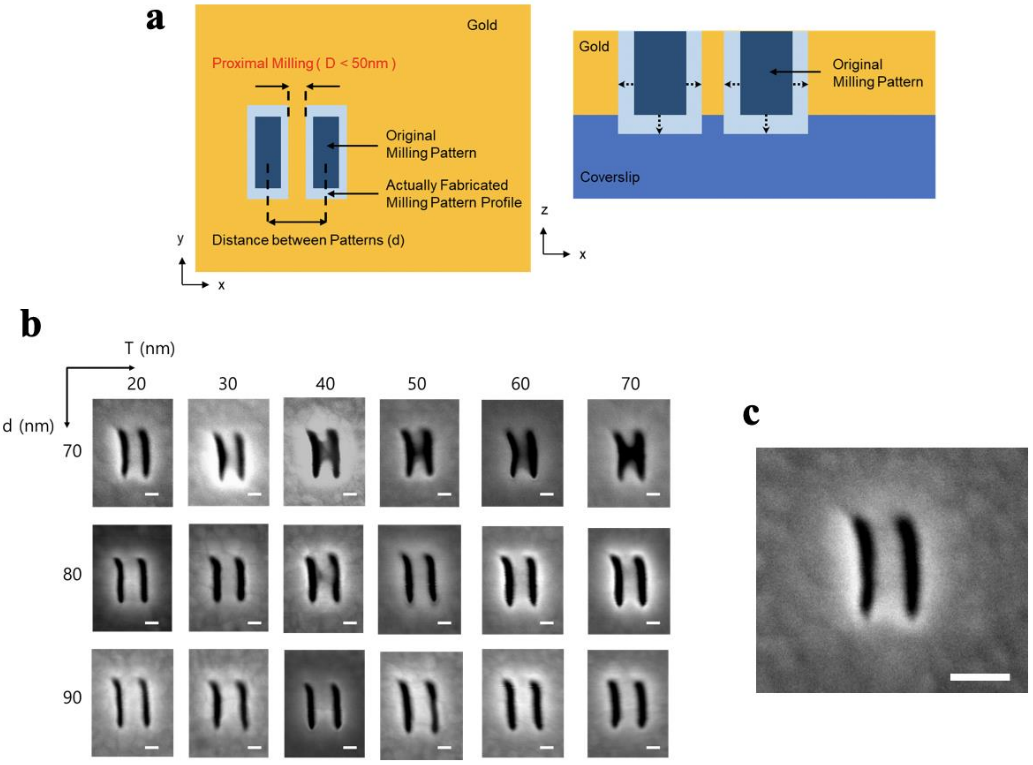

Supplementary Figure 12. Fabrication of double nano-slits with sub-50-nm spacing. a and **b,** Proximal focused-ion-beam (FIB) milling techniques. By controlling the distance *d* between double nano-slits and the milling time *T*, the FIB resolution limit is improved. **c,** Fabricated double nano-slits with sub-50-nm spacing. Scale bars, 100 nm.

To fabricate the double nano-slits with sub-50-nm spacing, we employed proximal milling techniques in Ga^+-based focused ion-beam (FIB) processes (model: Quanta2003D) on sputtered gold film on a silica coverslip. We intentionally off-designed the milling patterns from the original double nano-slit design to make use of the proximity effect of FIB milling, as shown in Supplementary Figure 12a. By optimally controlling the distance (*d*) between two rectangular milling patterns, we could fabricate sub-100-nm spacing between nano-slits, as shown in Supplementary Figure 12b. Additionally, we controlled milling time (*T*) to optimize the proximity. With these optimizations, we could overcome the resolution limit (~100 nm) of the conventional FIB milling and successfully fabricated the sub-50-nm spacing double nano-slits, as shown in Supplementary Figure 12c. A 100-nm-thick gold film was sputtered on a quartz substrate with the evaporation rate of 1.67 Å/s. The purity of the gold used for evaporation exceeded 99.99%. The root-mean-square and peak-to-peak roughness of the deposited gold film were measured to be 0.7 nm and 5 nm, respectively, by atomic force microscopy.
